# Post-arthroscopic osteonecrosis of the medial tibial plateau: a case series

**DOI:** 10.1186/s13256-016-1063-8

**Published:** 2016-10-19

**Authors:** Axel Marx, Alexander Beier, Pouria Taheri, Martin Röpke, Thomas Kalinski, Andreas M. Halder

**Affiliations:** 1 Clinic for Orthopaedics, Sana Kliniken Sommerfeld, 16766 Sommerfeld, Germany; 2 Clinic for Orthopaedics, University Magdeburg, 39120 Magdeburg, Germany; 3 Department of Pathology, University Magdeburg, 39120 Magdeburg, Germany

**Keywords:** Osteonecrosis, Tibial plateau fracture, Arthroscopy, Osteoporosis

## Abstract

**Background:**

Avascular necrosis after arthroscopic surgery of the knee has already been published. The purpose of this article is to report on the frequently misdiagnosed entity of osteonecrosis of the medial tibial plateau.

**Case presentation:**

Charts and radiographs of a consecutive series with isolated medial tibial plateau osteonecrosis were analyzed. The criterion for inclusion was the absence of trauma. Six caucasian female patients with an average age of 76.5 years complied with this criterion. Three of these cases had had arthroscopic intervention for medial meniscal lesion within the previous year.

**Conclusions:**

The etiology of these necroses remains unclear. Osteonecrosis must be taken into account as a possible cause of persistent knee pain after surgery. Correlation between arthroscopic treatment and necrotic processes in the tibial plateau must still be regarded with skepticism.

## Background

Pain in the medial compartment of the knee is a common symptom in orthopedic practice. A number of possible causes must be considered and therefore differential diagnosis is important. If trauma can be excluded, the discomfort may be due to various degenerative conditions (Table 1).

**Table 1:** Differential diagnosis for medial compartment knee pain

**Differential diagnosis**	
Medial meniscus lesion	
Medial and/or femoropatellar osteoarthritis	
Medial femur condyle osteonecrosis (M. Ahlback)	
Synovitis	
Intra-articular free bodies	
ACL/PCL instability and medial collateral ligament lesion	
Injury of the pes anserinus	
Medial plica shelf syndrome	
Referred pain from hip joint or lower lumbar spine disorders	

*ACL* anterior cruciate ligament, *PCL* posterior cruciate ligament

### Osteonecrosis of the knee

Spontaneous osteonecrosis (SPONK) of the medial femoral condyle was reported by Ahlback *et al.* in 1968 [1]. The exact etiology of this condition is still unclear, but SPONK is considered to be probably due to mechanical causes. Furthermore, correlation between meniscal tears and necrosis of the condyles is documented [2–4]. Spontaneous osteonecrosis is mainly found in the medial compartment of the knee and the defect areas are smaller than in secondary diseases [5, 6].

Secondary osteonecrosis (SONK) is associated with certain risk factors such as corticosteroid medications, alcohol abuse or various metabolic predisposing factors [6–13]. Various joints can be affected by SONK and bilateral defects are seen in 30–80 % of cases [5, 13, 14].

Patients diagnosed with SONK are generally slightly younger than those with SPONK (SONK: < 55 years versus SPONK > 60 years), and women are more often affected by both conditions than men [15].

Little is known about osteonecrosis of the tibial plateau. Only 2 % of osteonecrosis around the knee may affect the tibial plateau [16]. Spontaneous medial tibial plateau osteonecrosis was first described by D‘Angelijan in the mid-seventies [17, 18]. It was seen to be associated with minor arthritis of the knee and also related to minimal trauma or an increased activity level [18]. The medial tibial plateau is more frequently affected than the lateral [18, 19], and the extent of the defects may be significantly influenced by leg axis deformity [16]. As with osteonecrosis of the femoral condyle, primary and secondary necrosis of the tibial plateau can be differentiated. Exact values for these conditions are not available in the literature.

There are no specific clinical symptoms for spontaneous and secondary osteonecrosis of the tibial plateau, but synovitis and small effusions are often found [20]. The patients report tenderness on the medial tibia, below the joint line [18].

We report on our experience with osteonecrosis of the medial tibial plateau as a rare differential diagnosis for medial knee pain. The purpose of this article is to discuss a possible correlation between surgical intervention and the natural history of this type of osteonecrosis.

## Case presentations

Six caucasian cases of nontraumatic medial tibial plateau osteonecrosis with fracture of the cortical rim were treated in our department within a 12-month period. We included only cases with isolated tibial osteonecrosis in this survey. Cases with combined defects, for example, a combination with femoral condyle osteonecrosis, were excluded. Furthermore, the study included only cases with an uneventful history for trauma or ligament lesions and who had not undergone any knee or hip surgery prior to the index procedure. A standardized analysis of the biographical data, charts and operation reports was performed.

The patients underwent intensive examination for diseases and predisposition for vascular insufficiency. Clinical examination and standardized radiographs (one-leg, weight-bearing anterior-posterior and lateral, patella axial and full-leg axis) were performed in all cases. Bone quality was evaluated by dual energy X-ray absorptiometry (DEXA) testing (GE Medical Systems, Waukesha, WI, USA). The results of these examinations are presented in Table 2.

**Table 2:** Clinical data of the patients

No.	Patient	Age (Y)	Gender (F/M)	Ethnicity	BMI	DEXA *t* value	ID	Medication	AS	Interval AS to ON	Radiography	Subsequent surgery
1	Case 1 patient	80	F	Caucasian	25.6	−2.5	COPD	Corticosteroids	MPR	8 months	Mild OA, ON/TP	Postponed
2	Case 2 patient	78	F	Caucasian	22.8	−2.6	–	Biphosphonate	MPR, CP	9 months	Mild OA, ON/TP	TKA
3	Case 3 patient	88	F	Caucasian	18.1	−4.3	–	Biphosphonate	–	6 months	Mild OA, complex TP #	TKA
4	Case 4 patient	70	F	Caucasian	30.8	n.n.	–	–	–	–	Mild OA, ON/TP	Postponed
5	Case 5 patient	73	F	Caucasian	20.6	−3.2	RA	Corticosteroids	–	–	Mild OA, ON/TP	TKA
6	Case 6 patient	70	F	Caucasian	27.0	−1.0	–	–	MPR, CP	–	Mild OA, ON/TP	TKA

**Fig. 1:**
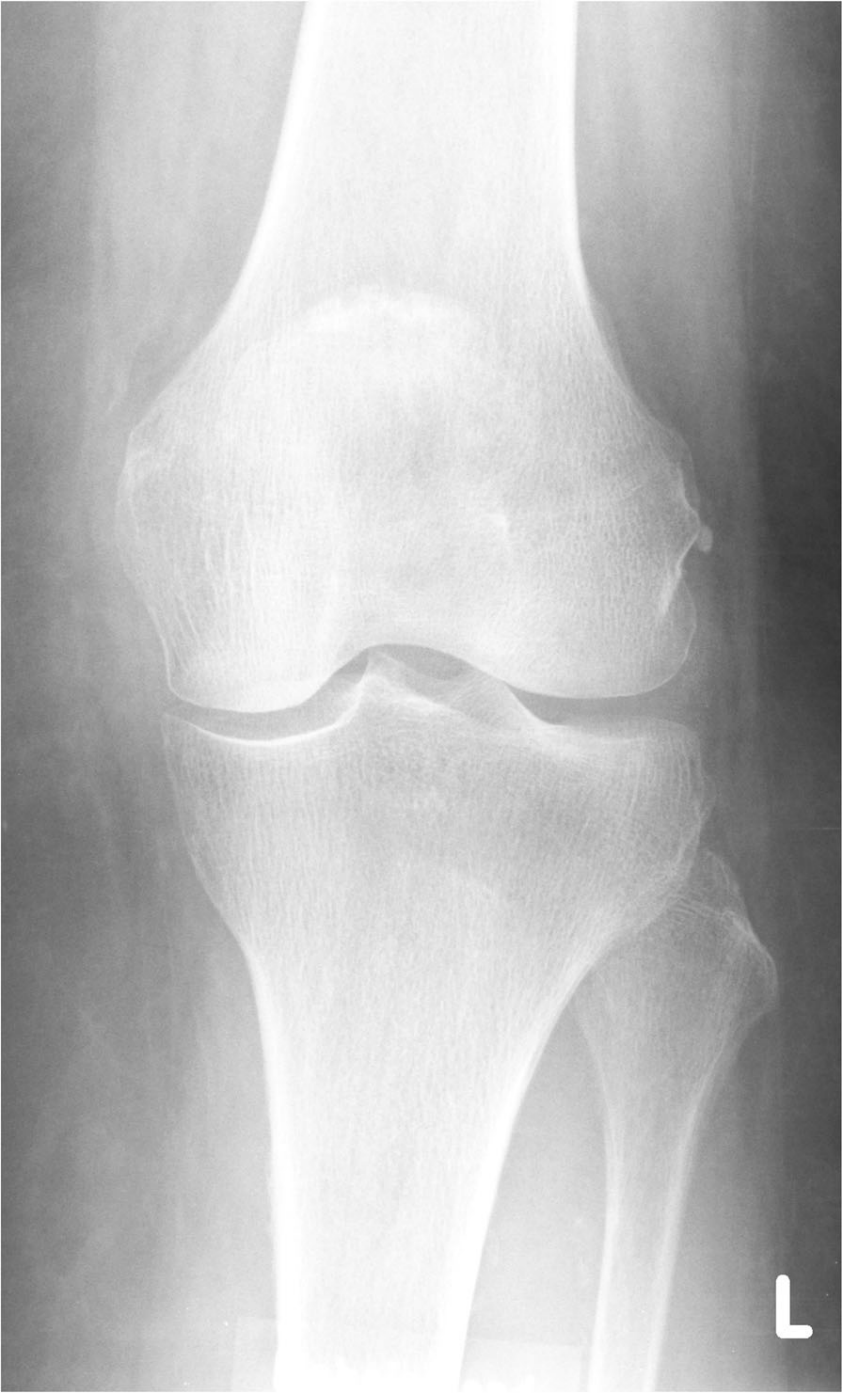
Case 1 patient: anterior-posterior radiography left knee: minimal medial joint space narrowing, absence of tibial osteophytes

**Fig. 2:**
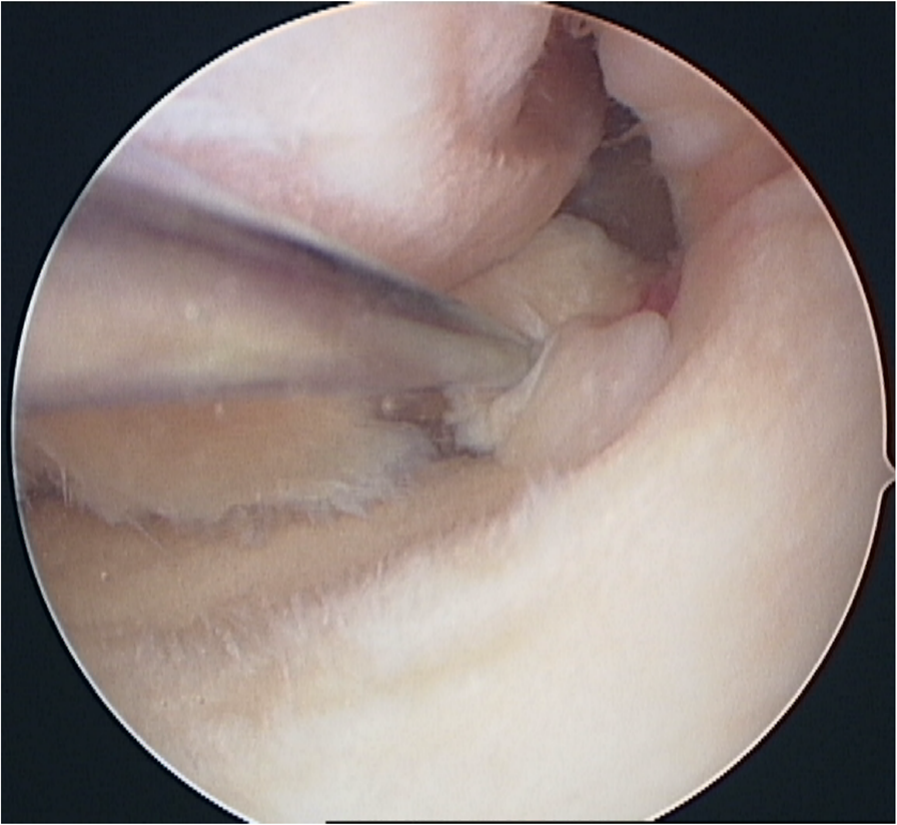
Case 1 patient: arthroscopic view: posterior radial meniscal lesion due to the meniscal base, moderate cartilage lesion tibial (grade 2)

*Y* years, *F/M* female/male, *BMI* body mass index, *DEXA* dual energy X-ray absorptiometry, *ID* internal diseases, *COPD* chronic obstructive pulmonary disease, *RA* rheumatoid arthritis, *AS* arthroscopic treatment, *MPR* meniscus partial resection, *CP* chrondroplasty, *ON* osteonecrosis, *OA* osteoarthritis, *TP* tibia plateau, *TP #* tibia plateau fracture, *TKA* total knee arthroplasty;

All six patients were women and their average age was 76.5 years (70–88 years). Initial radiographs presented minimal medial osteoarthritis (stage 1 according to Kellgren and Lawrence [21], Fig. 1). One-leg axis under weight-bearing presented mild varus deformity in all the patients. There were no signs of trauma or osteonecrosis in the first radiographic assessment. None of the cases had sclerosis or osteophytes on the condyles or tibial plateau. All radiographies were analyzed by AM. The patients were initially treated by conservative methods such as physiotherapy and nonsteroidal medication for a certain period. Three patients had had arthroscopic treatment due to ongoing discomfort (case 1, 2, and 6 patient). The indication for surgery in these cases was medial meniscus lesion, and arthroscopic medial partial meniscectomy was performed (Fig. 2). There was no treatment of the tibial cartilage (for example, micro-fracturing or drilling). The early postoperative period was uneventful in all cases.

**Fig. 3:**
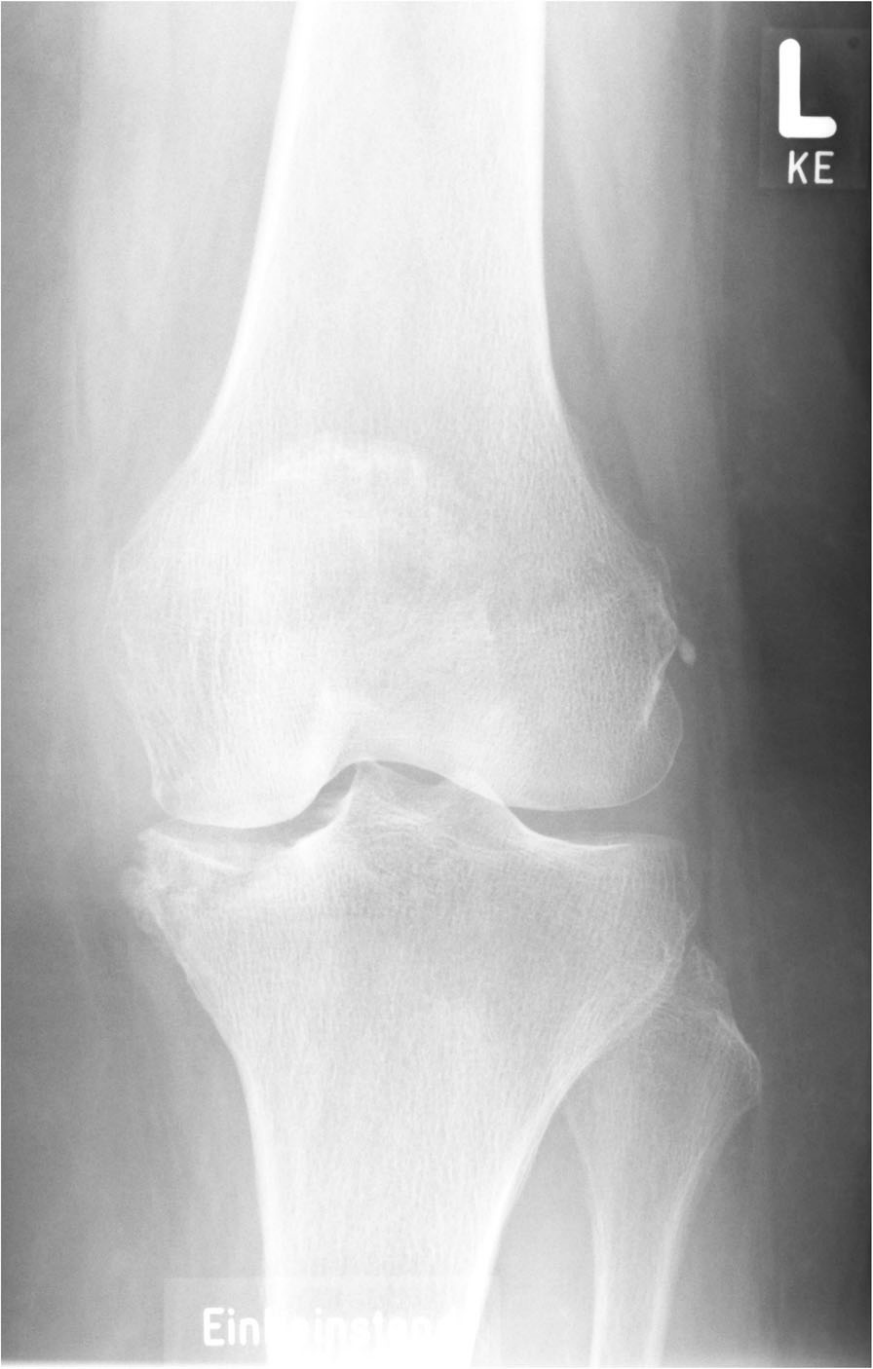
Case 1 patient: anterior-posterior radiography left knee: medial tibial plateau osteonecrosis, cystic demarcation. L = left, KE is no information concerning the patient

Persistent discomfort after rehabilitation led to further investigations in our department. Repeat radiographs showed isolated osteonecrosis of the medial tibial plateau with fracturing of the cortical tibial rim (case 1 patient, Fig. 3). The average interval between index surgery and identification of the medial tibial plateau osteonecrosis was 7.6 months. In all three cases (case 1, 2, and 6 patient) the medial compartment (corresponding to the partial meniscectomy) was affected.

In the three non-operated cases (case 3, 4, and 5 patient) the average interval between the occurrence of the first symptom and diagnosis of the osteonecrosis was 5 months (Table 3). The radiographies presented an identical isolated necrosis of the medial tibial plateau with a fractured cortical rim.

**Table 3:** Interval symptom to treatment

No.	Patient	Interval symptom to AS	AS	Interval AS to ON	Interval symptom to ON
1	Case 1 patient	3 weeks	MPR	8 months	–
2	Case 2 patient	1 year	MPR, CP	9 months	–
3	Case 3 patient	–	–	–	2 months
4	Case 4 patient	–	–	–	9 months
5	Case 5 patient	–	–	–	4 months
6	Case 6 patient	6 months	MPR, CP	6 months	–

*AS* arthroscopic treatment, *MPR* meniscus partial resection, *CP* chrondroplasty, *ON* osteonecrosis

The radiographic staging of the defects was adapted to Carpintero [16], Lotke [18] and the modified Ficat and Arlet classification for the knee [22] (Table 4). All six cases were classified as stage 3 to 4 (Table 4).

**Table 4:** Staging of osteonecrosis based on planar radiography, scintigraphy, and magnetic resonance imaging

Stage	Radiography	Scintigraphy	Magnetic resonance imaging
1	Normal	Increased uptake	Relatively small and well localized, low signal in subchondral zone (T1)
2	Abnormal, cystic and sclerotic changes	Increased uptake	Low signal area subchondral zone diffused down to metaphysis
3	Crescent sign and subchondral collapse producing crescent or rim sign	Increased uptake	Changes with widespread diffusion in metaphysis, surrounded by reactive bone rim,
4	Arthritic changes, joint narrowing with or without condylar involvement	Increased uptake	Diffuse areas of abnormal marrow signal intensity, involvement of the condyle possible

**Fig. 4:**
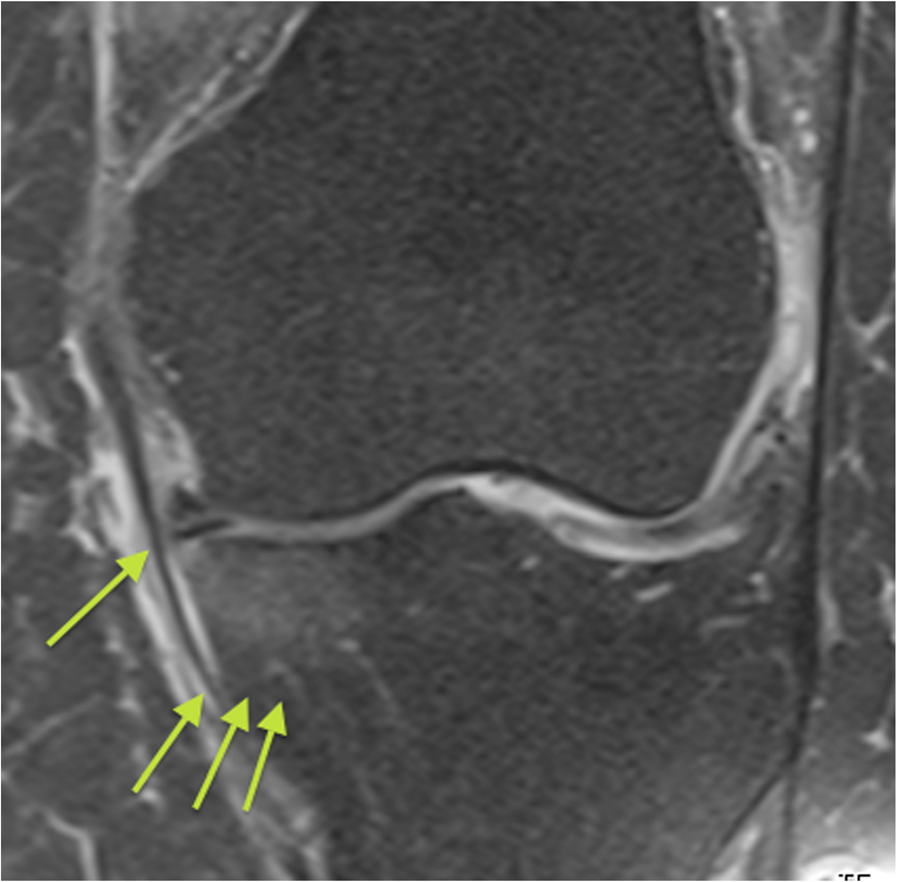
Case 1 patient: preoperative T2-weighted magnetic resonance imaging left knee: slight edema of the medial tibia plateau, medial meniscus lesion. The single *arrow* points to the meniscus lesion, the group of three *arrows* point to the edema of the medial tibial plateau

A magnetic resonance imaging (MRI) scan of case 1 patient is presented in Fig. 4. The preoperative MRI showed the lesion of the medial meniscus as well as concomitant slight edema of the medial tibial plateau.

**Fig. 5:**
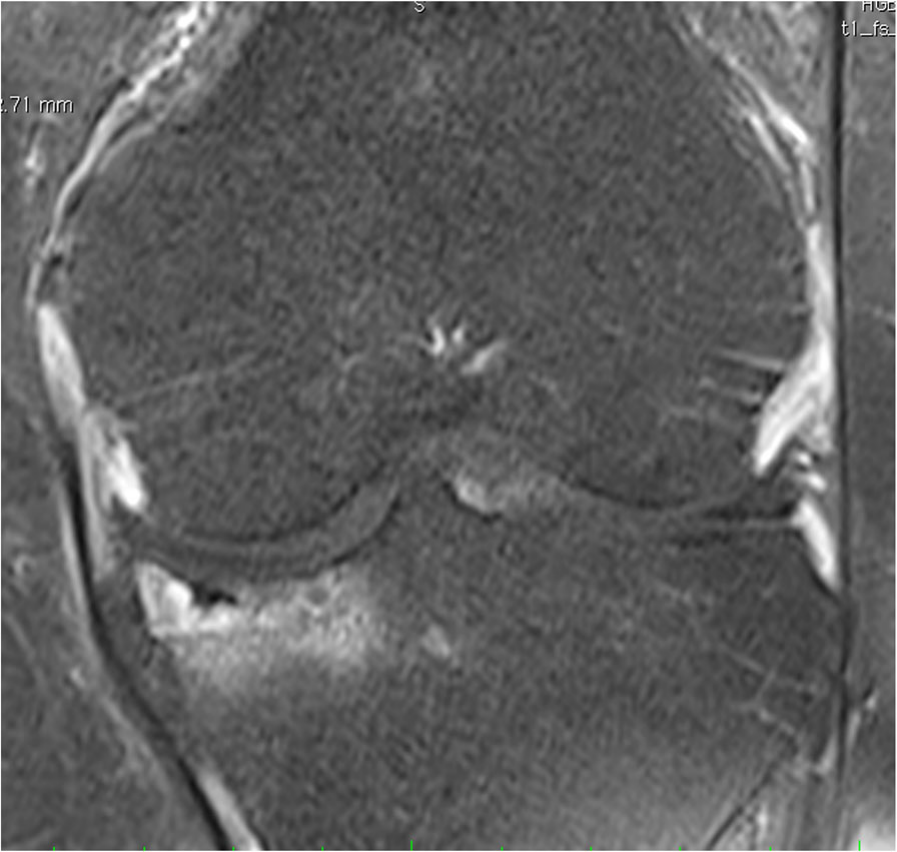
Case 1 patient: postoperative T2-weighted magnetic resonance imaging left knee: progression of the tibial edema, cystic defects and depression of the medial tibia plateau

A further MRI scan after arthroscopic treatment (case 1 patient) revealed the ongoing tibial process and a cystic necrotic bone defect in the medial tibial plateau (Fig. 5).

**Fig. 6:**
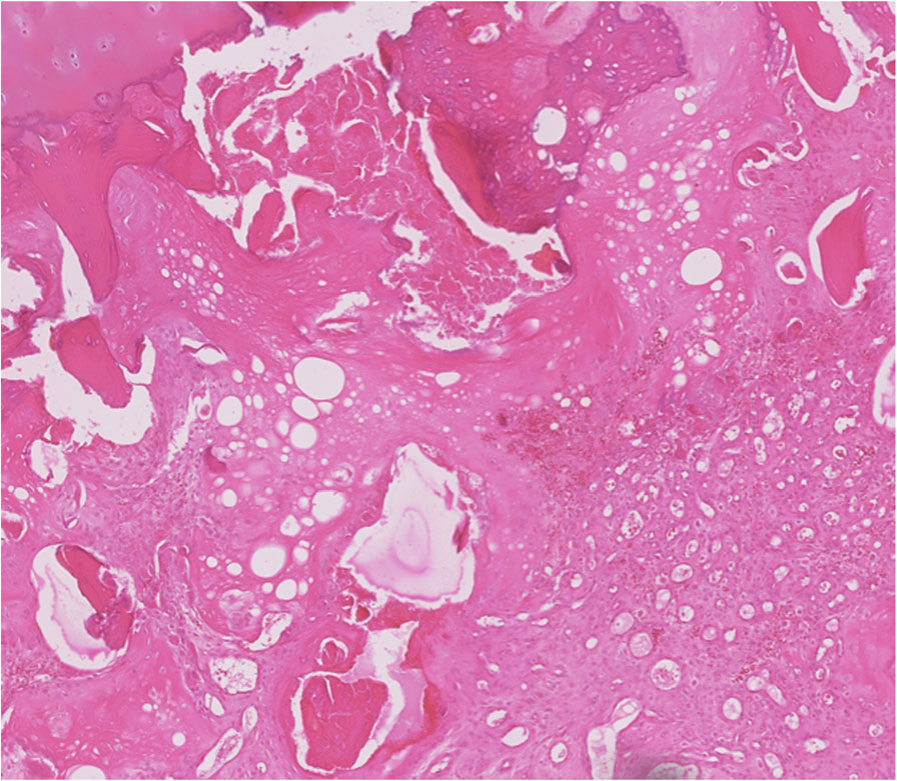
Case 2 patient: histological image: osteonecrosis with secondary infraction showing necrotic debris surrounded by callus-like capillary-rich granulation tissue

All six patients were scheduled for total knee arthroplasty. Histological analyses were performed on case 2 patient (Fig. 6) and osteonecrosis with secondary infarction of the medial plateau was detected. No infection or malignancy was seen.

## Discussion

### Is spontaneous osteonecrosis really spontaneous?

There are various theories regarding the genesis of these “spontaneous osteonecroses” [9]. The existence of these numerous cases of spontaneous defects of the knees has been doubted [23]. In most cases, the reasons for the ischemic bone deaths were identified and a high correlation between meniscus tear/osteoarthritis and osteonecrosis is documented [2, 23]. Defects on the subchondral bone plate caused by chronic stress or minor trauma lead to subchondral microfracture and vascular disturbance of the bone underneath, resulting in osteonecrosis [5–9, 24, 25]. In particular, radial meniscus lesions and posterior root tears are identified as highly correlative with osteonecrosis of the femoral condyle [9, 23, 26].

In cases of partial meniscectomy the limited buffer creates high stresses which may initiate the ischemic process [2, 3, 9, 10]. On the other hand, a high incidence of meniscal lesions is documented in asymptomatic knees [27]. An MRI analysis by Navarro *et al*. revealed that a meniscus lesion had led to osteonecrosis on the ipsilateral side in about 57 % [28].

In the recent literature the new term, “osteoporotic subchondral insufficiency/stress fracture”, has been redefined [20, 23, 29]. The intention was to replace the word “spontaneous”, therefore the subchondral fracture is named as a promoter of the disease. As early as the 1980s Manco *et al*. reported on five cases of insufficiency fractures of the tibial plateau [30]. Here, osteoporotic bone had caused fracturing of the underlying bone. Lotke *et al.* concluded that local stress leads to subchondral injuries and edema. Necrosis may be initiated by increasing marrow pressure [18], then repeated impact on the osteopenic or osteoarthritic bone stock leads to these microfractures. A raised activity level could also be responsible for progression of the disease. After histological analysis of fourteen cases, Yamamoto and Bullough stated that osteonecrosis was a result of a subchondral insufficiency fracture [6]. Here, the defects were located only in the femoral condyle. No isochronous meniscus lesions were specified.

### Treatment options

Stage-dependent treatment is recommended. In the early stages (1 and 2), conservative treatment is still possible and may be helpful, depending on the size of the defects and the integrity of the cortical rim [29, 31]. The use of crutches to reduce weight-bearing can help to avoid complete collapse of the compartment [31]. Additional medication with Alendron, 10 mg per day for a month, is useful [32].

As a joint-preserving procedure, ante- or retrograde drilling can be an option to induce reperfusion of the necrotic bone area [8]. Otherwise, the results are only encouraging in the early stages (1 and 2) [22, 33]. In stages 2 and 3, high tibial osteotomies can be performed to reduce pressure on the weakened bone area [34]. Finally, implantation of a uni- or bicondylar knee replacement is the procedure which leads to the most satisfactory outcome in the treatment of persisting knee pain [20, 29, 35]. In cases of secondary osteonecrosis total knee arthroplasty seems to be more successful than hemiarthroplasty. In older patients, pre-existing osteoarthritis and defects in the cortical rim are indications for knee replacement surgery.

### Post-arthroscopic osteonecrosis

During the last decade several reports have been published on “post-meniscectomy osteonecrosis” of the femoral condyle [2–4, 8, 10, 36–38]. Meniscus and cartilage surgery is the common treatment in all these publications. These case series are small. Laser surgery for meniscal and chondral lesion or extensive drilling was identified as a possible cause [2, 39]. Garino *et al*. reported on five patients suffering from laser-induced osteonecrosis in the area of treatment [39].

Most of the published case series were retrospective and pre-existing osteonecrosis could not be excluded by preoperative diagnostics [8, 10, 37]. A four- to six-week diagnostic window to detect initial osteonecrosis is needed [10]. In preoperative MRI this time period between the onset of pain and the MRI diagnostics should be respected in order not to miss the initial stage of the pathology [2, 10, 40]. Johnson *et al*. presented a case series of seven patients with post-arthroscopic osteonecrosis of the knee [2]. This is the only study with a consistent minimum period of six weeks between the onset of knee pain and the MRI diagnostics. All MRIs were documented with no signs of osteonecrosis before surgical treatment. The postoperative necrosis was located in the medial condyle in four cases, in the lateral condyle in one case and in both the medial and lateral tibial plateau in the two remaining cases. The population had an average age of 60 years. A direct correlation between arthroscopy and outcome remains speculative.

Direct correlation between arthroscopic meniscus surgery and osteonecrosis of the tibial plateau cannot be proven by retrospective analysis. Inadequate surgical skill is a risk to the integrity of the femoral condyles, but plays no role in defects of the tibial plateau. It is almost impossible to cause iatrogenic damage in this area. The natural history of tibial plateau osteonecrosis may differ from that of femur condyle necrosis. In our collective, all the patients were older and female, which leads us to the opinion that gender and age are predicting factors [16]. Osteopenic bone stock is common in older female patients. DEXA analysis confirms this hypothesis in our group. In one case (case 5 patient) the periarticular osteoporosis was additionally influenced by rheumatoid arthritis.

Whether surgery had any direct influence remains unclear. Three patients with no prior surgical intervention on the knee suffered an identical tibial plateau osteonecrosis.

**Fig. 7:**
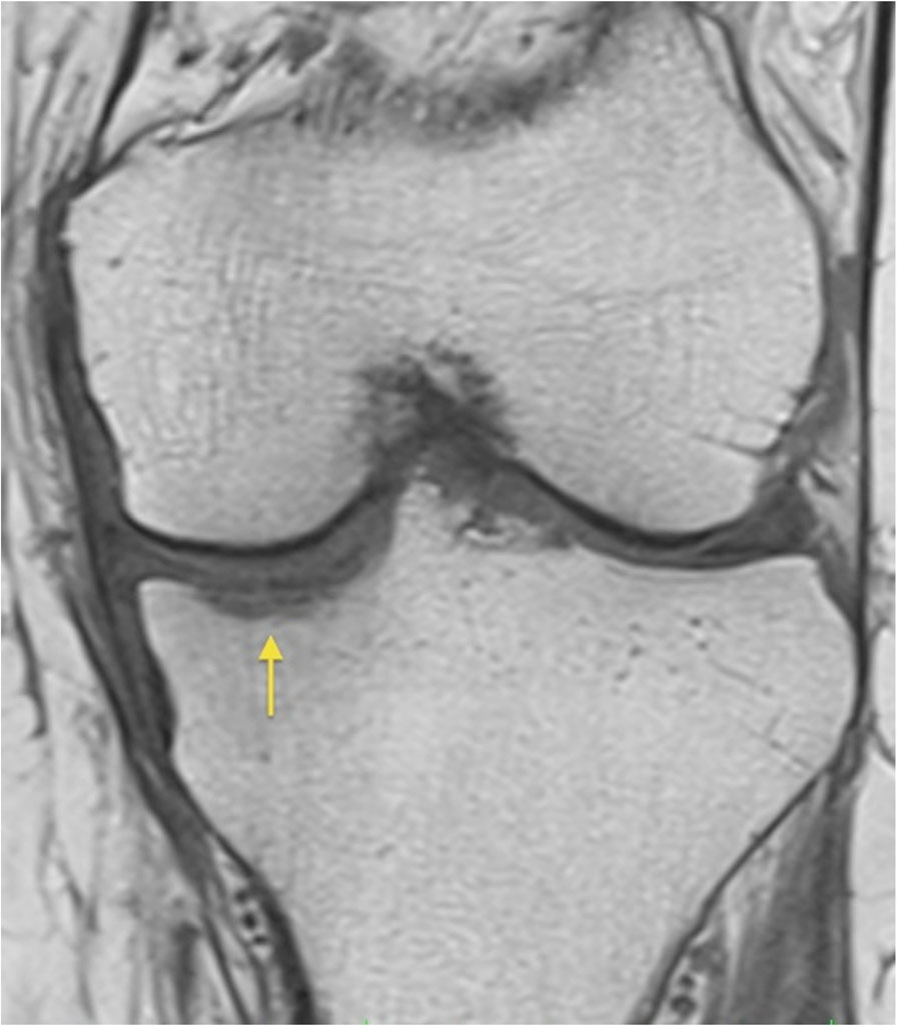
Case 1 patient: preoperative T1-weighted magnetic resonance imaging left knee: underneath the articular surface linear subchondral insufficiency fracture (*arrow*)

In the presented MRI the subchondral insufficiency fracture can already be identified preoperatively (Fig. 7). This supports the theory that osteonecrosis in the medial tibial plateau may be a physician-independent disease of the knee in older, female, and osteoporotic patients. Further investigation is necessary to prove this hypothesis.

In our management of these older female cases, preoperative MRI has been made obligatory to prevent medico-legal discussion in this population group.

## Conclusions

Osteonecrosis of the tibial plateau should be considered when older osteoporotic patients complain of knee pain. Even re-assessment of primarily physiological radiographs using MRI and a new X-ray may help to identify the correct cause of the discomfort.

## Abbreviations

ACL: anterior cruciate ligament
AS: arthroscopic treatment
BMI: body mass index
COPD: chronic obstructive pulmonary disease
CP: chrondroplasty
DEXA: dual energy X-ray absorptiometry
F/M: female/male
ID: internal diseases
MRI: magnetic resonance imaging
MPR: meniscus partial resection
OA: osteoarthritis
ON: osteonecrosis
PCL: posterior cruciate ligament
RA: rheumatoid arthritis
SONK: secondary osteonecrosis
SPONK: spontaneous osteonecrosis
TKA: total knee arthroplasty
TP: tibia plateau
TP #: tibia plateau fracture
Y: years
